# Prehospital glucagon-like peptide-1 receptor agonists are not associated with reduced inpatient opioid exposure but with shorter hospital length of stay after total joint arthroplasty

**DOI:** 10.3389/fendo.2026.1918370

**Published:** 2026-08-21

**Authors:** Noam Vaknin, Chen Seidenberg, Eric Sitton, Pierre Singer, Ruth Mishali, Hila Vidal, Michal Slevin-Kish

**Affiliations:** 1 Herzliya Medical Center, Herzliya, Israel; 2 Cancer Research Center and Wohl Institute for Translational Medicine, Tel Hashomer, Ramat Gan, Israel; 3 Gray Faculty of Medicine and Health Science, Tel Aviv University, Tel Aviv, Israel; 4 Critical Care Medicine and Institute for Nutrition Research, Rabin Medical Center, Beilinson Hospital, Petah-Tikva, Israel; 5 The Research Lab on Computerized Decision Making, School of Medicine, Reichman University, Herzliya, Israel

**Keywords:** GLP-1 receptor agonists, length of stay, opioid exposure, osteoarthritis, postoperative pain

## Abstract

**Aims:**

Glucagon-like peptide-1 receptor agonists (GLP-1RAs) have been hypothesized to confer anti-inflammatory, antinociceptive, and anti-addictive benefits. We aimed to investigate their impact on acute perioperative pain and opioid use after total joint arthroplasty (TJA).

**Methods:**

A retrospective cohort study analyzed 14,375 hip or knee arthroplasties performed between January 2018 and February 2026, which included 882 patients on chronic preoperative GLP-1RA therapy. Using 1:1 propensity-score matching without replacement, 786 GLP-1RA users were matched to 786 non-users (total n=1,572) based on demographics, surgery type, and chronic medication use.

**Results:**

In the matched cohort, chronic GLP-1RA use was not associated with reduced inpatient opioid administration (adjusted difference −0.18; 95% CI, −0.74 to 0.38; p=0.53) or corrected postoperative morphine milligram equivalents (MME) (−2.79 MME; 95% CI, −6.95 to 1.37; p=0.19). When normalized to hospitalization time, the difference in inpatient opioid exposure was not statistically significant (+0.073 MME/hour; 95% CI, −0.003 to 0.149; p=0.059). Average visual analog scale (VAS) pain scores also showed no significant difference (−0.094; p=0.15). However, GLP-1RA users demonstrated a consistently shorter length of stay (LOS) (adjusted difference −10.41 hours; 95% CI, −13.97 to −6.85; p<0.001).

**Conclusions:**

Chronic preoperative GLP-1RA therapy was not associated with reduced inpatient opioid exposure or improved early postoperative pain control following TJA. Nevertheless, GLP-1RA use was consistently associated with shorter hospital LOS. Prospective studies are needed to determine whether this finding reflects improved recovery pathways or residual confounding and to evaluate the impact of GLP-1RAs on longer-term postoperative outcomes and opioid utilization.

## Background

Osteoarthritis (OA) is a leading cause of global disability, characterized by chronic pain, functional impairment, and progressive joint degeneration (1). Total joint arthroplasty (TJA) remains the definitive treatment for end-stage OA (2); however, managing acute postoperative pain and minimizing opioid consumption continue to be significant clinical challenges (3). a 2021 systematic review demonstrated that more than 1 in 5 patients had continued opioid use that was sustained for 12 months postoperatively (4).

Obesity and type 2 diabetes mellitus (T2DM) are major risk factors that not only accelerate OA progression but also complicate perioperative recovery, often leading to increased opioid requirements and prolonged hospital stays (5).

Glucagon like peptide-1 receptor agonists (GLP-1RAs) emerged as potential disease modifying agents in OA (DMOADs) (6). Beyond their systemic effects on glycemic control and weight reduction, GLP-1RAs exhibit direct anti-inflammatory and chondroprotective properties (7). In addition to their metabolic effects, GLP-1 receptors are expressed in human chondrocytes, where their activation suppresses matrix metalloproteinase activity and reduces inflammatory mediators including IL-6, IL-8, and TNF-α via NF-κB pathway inhibition (8, 9).

Emerging evidence highlights the neuroprotective and antinociceptive potential of GLP-1 RAs, demonstrating benefits in neurodegenerative diseases and pain disorders through anti-inflammatory, antioxidant, and endogenous opioid mediated mechanisms, potentially offering a novel mechanism for reducing postoperative pain intensity (10). Furthermore, recent clinical and preclinical data from early 2026 suggest that GLP-1RAs possess significant anti addictive properties by modulating the brain’s reward system, specifically the dopaminergic pathways in the ventral tegmental area. This effect has been shown to potentially reduce the craving and consumption of various substances, including alcohol and tobacco, raising the possibility that GLP-1RA therapy could also mitigate the risk of postoperative opioid escalation or long-term dependency (11, 12).

Clinical evidence regarding the perioperative benefits of GLP-1RAs in orthopedic surgery is growing. Recent meta-analysis of 346,899 patients after knee and hip replacements demonstrated a lower 90-day risk prostatic joint infection following total knee replacement, and lower readmission and revision rates with GLP1 use (13).

However, the significant weight loss associated with GLP-1RA therapy often includes a reduction in lean body mass (14). This shift in body composition is a critical consideration in the surgical setting, as maintaining muscle mass is essential for functional recovery and postoperative mobility (15).

The direct impact of chronic GLP-1RA use on immediate postoperative pain trajectories and opioid consumption in the TJA population, particularly given the interplay between neuroprotection, reward system modulation, and muscle mass has not been definitively characterized. While some data from oncological cohorts suggest limited effects on acute pain (16), the specific metabolic and inflammatory profile of arthroplasty patients warrants further investigation.

Although opioid consumption is frequently used as a surrogate outcome in studies evaluating perioperative interventions, inpatient opioid administration following TJA is strongly influenced by real-world clinical practice (3, 5). Standardized multimodal analgesic protocols, nursing-driven pain assessment and medication administration, enhanced recovery pathways, early mobilization requirements, and discharge criteria all contribute to postoperative opioid exposure (3, 5). Consequently, inpatient opioid utilization reflects not only individual pain intensity but also institutional care pathways and rehabilitation protocols. Furthermore, many patients undergoing TJA for osteoarthritis have substantial analgesic exposure before surgery, and preoperative analgesic or opioid use is one of the strongest predictors of continued postoperative analgesic consumption (4). Large real-world cohort data have also shown that obesity, greater comorbidity burden, and knee arthroplasty are associated with higher postoperative opioid use (5).

In this study, we utilized a large inpatient cohort of 14,375 patients undergoing elective joint replacement, of whom 882 were managed with chronic GLP-1RA therapy. We aimed to evaluate the association between preoperative GLP-1RA use and inpatient postoperative pain trajectories and opioid requirements. By analyzing this significant cohort, we seek to determine whether the anti-inflammatory, neuroprotective, and anti-addictive benefits of GLP-1RAs translate into superior acute recovery in the immediate postoperative period.

## Methods

This retrospective cohort study was conducted at Herzliya Medical Center, a high-volume elective surgical center. Consecutive patients who underwent total hip or knee arthroplasty between January 2018 and February 2026 were included. The analytic cohort comprised 14,375 arthroplasty surgeries, including 882 surgeries among GLP-1 RA users and 13,493 surgeries among non-users.

Demographic and hospitalization data were extracted from institutional electronic medical records. Variables included age, sex, BMI, ASA physical status classification, surgery type, revision status, chronic medications use, including insulin use and non-insulin diabetic medication, opioid administrations, and additional analgesics like gabapentinoids, NSAIDs, visual analog scale pain scores (VAS), and length of hospital stay.

The exposure of interest was chronic GLP-1 RA use, identified from the chronic medication record. Surgeries were classified as GLP-1 RA exposed if GLP-1 RA use was documented before surgery. All other surgeries were classified as non-users.

GLP-1 RA exposure was ascertained from the chronic medication record completed in the pre-surgery clinic, which captured adherence, dose, treatment duration, indication, the specific GLP-1 RA agent, and the timing of the last dose before surgery. As part of this visit, patients received standardized verbal instructions on perioperative adjustment of their GLP-1 RA regimen, in parallel with counseling regarding other chronic medications. In most cases, one GLP-1 RA dose in the week before surgery was withheld in line with contemporary guideline-based recommendations (17).

Therefore, our analysis evaluates the impact of chronic preoperative GLP-1 RA treatment and its resulting baseline physiological and metabolic effects rather than the effects of active drug administration immediately prior to surgery.

Given the pharmacokinetic characteristics of these agents, we anticipated that residual drug exposure and associated physiologic effects would persist at the time of surgery. Semaglutide, for example, has an elimination half-life of approximately one week, such that steady-state plasma concentrations are generally achieved only after around five weeks of continuous therapy (18), and a comparable period is required for near-complete resolution of its pharmacodynamic effects following discontinuation.

Data regarding the specific GLP-1 RA agent prescribed were collected, and a subgroup analysis evaluating differences between agents was performed (**Supplementary Figure 1**).

The main outcomes were postoperative inpatient opioid use and length of stay. Inpatient opioid use included inpatient opioid administration count, corrected total morphine milligram equivalents (MME) based on CDC clinical practice guidelines 2022 (19), corrected postoperative MME (administrations during surgery were omitted), and MME per length-of-stay hour. Length of stay was analyzed in hours and reported in days.

Valid VAS scores were defined as numeric values from 0 to 10 inclusive, with VAS = 0 retained as a valid score. Missing VAS values were not imputed, values outside 0–10 were excluded, and negative-time, missing-time were excluded. Exploratory pain outcomes included average VAS, maximum VAS, and any VAS score ≧7.

### Statistical analysis

All analyses were performed using R version 4.4.3. Continuous variables were summarized as means with standard deviations and medians with interquartile ranges. Categorical variables were summarized as counts and percentages.

Because GLP-1 RA users differed substantially from non-users before adjustment, propensity-score matching was performed to reduce measured confounding. A propensity score for GLP-1 RA exposure was estimated using age, BMI, chronic medication count, sex, ASA physical status classification, surgery type, revision status, and chronic opioid use. Model discrimination was assessed using the area under the receiver operating characteristic curve.

GLP-1 RA users were matched to non-users using 1:1 propensity-score matching without rematching. The matched cohort included 786 GLP-1 RA users and 786 matched non-users, yielding 1,572 surgeries and 786 matched pairs. Covariate balance before and after matching was assessed using standardized mean differences.

The primary analytic comparison was performed in the fixed matched cohort. Between-group differences were calculated as GLP-1 RA users minus matched non-users. Negative values therefore indicate lower values among GLP-1 RA users, and positive values indicate higher values among GLP-1 RA users. Adjusted models were used to estimate associations between GLP-1 RA use and postoperative outcomes in the fixed matched cohort.

The primary adjusted outcome models included GLP-1 RA exposure, BMI, a BMI-missing indicator, insulin use, non-insulin diabetes medication count, age, sex, surgery type, ASA score, chronic opioid use, pain-modulator use, psychiatric medication use, and chronic medications. Missing BMI and ASA values were handled using model-specific replacement and missingness indicators. Because the BMI- and ASA-missing indicators were perfectly collinear, only the BMI-missing indicator was retained in the final models. Standard errors were adjusted to account for clustering within matched pairs. Adjusted estimates were reported with 95% confidence intervals and p-values. Detailed specifications and estimates from the primary and reduced-covariate sensitivity models are provided in **Supplementary Table 1**.

As a sensitivity analysis addressing potential differences in healthcare access and financing, healthcare coverage insurance was classified as standard Health maintenance organization (HMO) insurance coverage, HMO insurance supplemental coverage (voluntary supplementary health services offered by Israeli health maintenance organizations in addition to the statutory health basket) or non-HMO insurance coverage, including commercial insurance, private payment, and international coverage. Healthcare insurance coverage category was added to the primary adjusted outcome models while preserving the fixed matched cohort and matched-pair clustered standard errors.

Postoperative day-binned trajectory analyses were performed descriptively in the matched cohort. Daily inpatient opioid exposure was summarized by postoperative day bins, including POD0, POD1, POD2, and POD3 +. POD3+ included postoperative day 3 and later while the patient remained hospitalized. Patients discharged before a later POD bin did not contribute to that bin. Because later POD bins are conditional on continued hospitalization, these analyses were interpreted descriptively.

Exploratory pain outcomes were analyzed separately from the main opioid and recovery outcomes. VAS assessment count was considered a measure of documentation and measurement frequency rather than a direct pain outcome. All statistical tests were two-sided, and p<0.05 was considered statistically significant. Pain outcomes and POD-binned trajectory analyses were considered exploratory.

### Ethical considerations

This study was approved by the institutional Helsinki Committee (IRB), approval number [0012-24-HMC]. Given the retrospective nature of the study and use of anonymized data, the requirement for informed consent was waived.

## Results

The analytic cohort comprised 14,375 arthroplasty surgeries, including 882 surgeries among GLP-1 RA users and 13,493 among non-users. Before matching, GLP-1 RA users differed substantially from non-users across several baseline characteristics, with the largest imbalances observed for diabetes, insulin use, non-insulin diabetic medication use, chronic medication use, BMI, and ASA physical status classification. Diabetes was present in 68.3% of GLP-1 RA users compared with 17.8% of non-users, and GLP-1 RA users had higher BMI and greater chronic medication burden. These baseline differences supported the use of propensity-score matching. The propensity-score model showed good discrimination for GLP-1 RA exposure, with an AUC of approximately 0.84.

**Figure 1:**
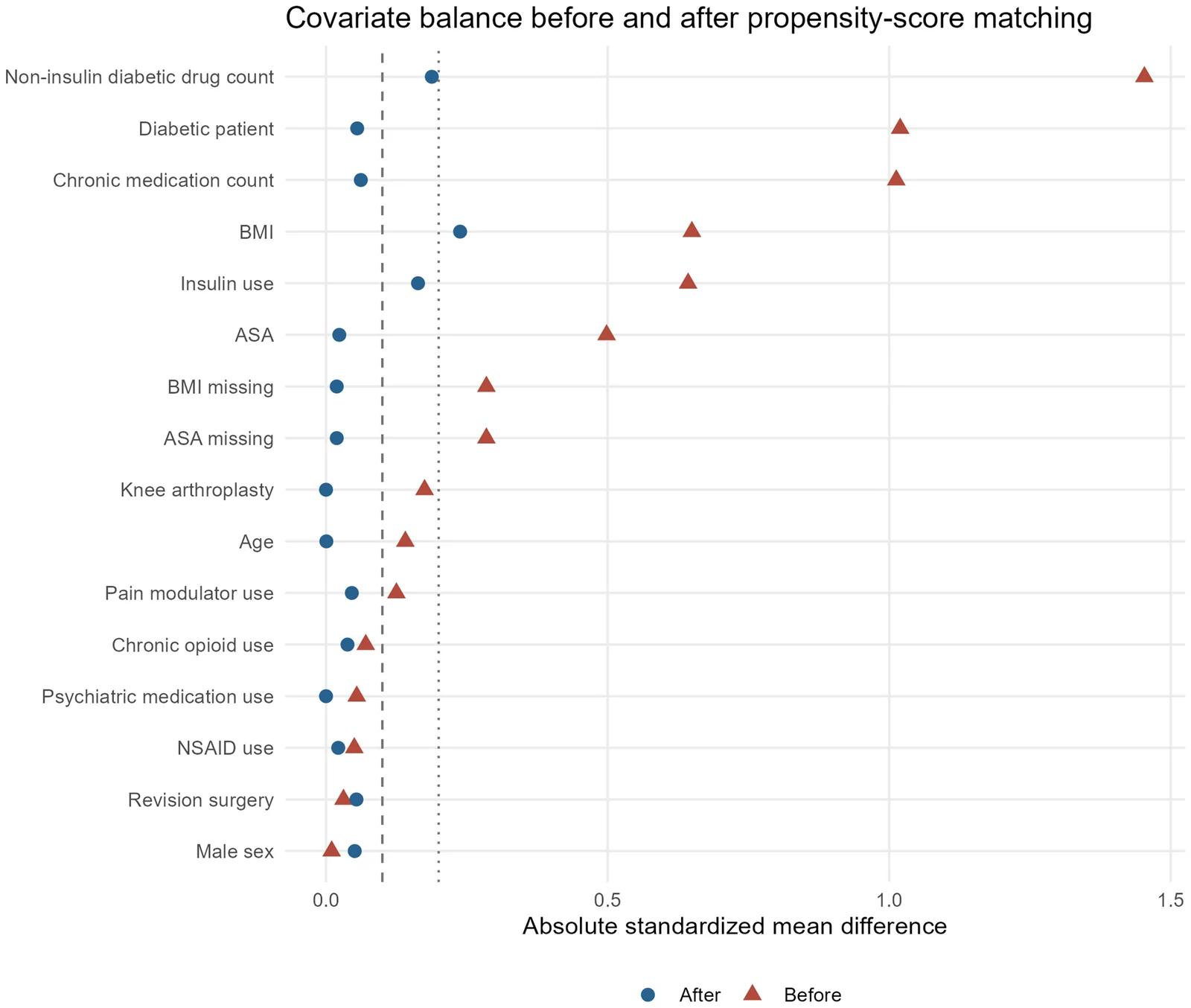
Covariate balance before and after propensity-score matching. Absolute standardized mean differences are shown for baseline covariates before and after 1:1 propensity-score matching of GLP-1 receptor agonist users to non-users. Red triangles indicate pre-matching imbalance and blue circles indicate post-matching imbalance. Vertical reference lines indicate absolute SMD thresholds of 0.10 and 0.20. Matching substantially improved covariate balance, although residual imbalance remained for BMI and diabetes-treatment variables. ASA, American Society of Anesthesiologists physical status; BMI, body mass index; NSAID, nonsteroidal anti-inflammatory drug; SMD, standardized mean difference.

After 1:1 propensity-score matching, the matched cohort included 786 GLP-1 RA users and 786 matched non-users, yielding 1,572 surgeries and 786 matched pairs. Matching substantially improved balance across most measured covariates, including age, sex, ASA physical status, surgery type, chronic medication use, psychiatric medication use, NSAID use, chronic opioid use, and gabapentinoids use. Residual imbalance remained for BMI, insulin use, and non-insulin diabetes medication use. Therefore, these variables were included as covariates in the final adjusted matched-cohort outcome models. Baseline characteristics before and after matching are shown in **Table 1**, and covariate balance is summarized in **Figure 1**.

**Table 1:** Baseline characteristics before and after propensity-score matcing.

	Analytic cohort (before matching)			Matched cohort (after matching)		
Variable	GLP-1 RA (n = 882)	Control (n = 13,493)	SMD	GLP-1 RA (n = 786)	Control (n = 786)	SMD
Age, years	68.99 ± 7.54; 70 [64, 74]	70.18 ± 9.26; 71 [65, 76]	-0.141	69.05 ± 7.51; 70 [64, 74]	69.05 ± 9.27; 70 [65, 75]	-0.001
*BMI, kg/m²*	33.6 ± 5.53; 33.2 [29.95, 36.65]	30.02 ± 5.49; 29.3 [26.3, 33.2]	**0.65**	33.73 ± 5.54; 33.3 [30, 36.75]	35.33 ± 7.78; 34.8 [29.78, 39.8]	**-0.238**
BMI missing	591/882 (67%)	10741/13493 (79.6%)	**-0.285**	527/786 (67%)	534/786 (67.9%)	-0.019
*ASA class*	2.68 ± 0.56; 3 [2, 3]	2.4 ± 0.55; 2 [2, 3]	**0.498**	2.68 ± 0.56; 3 [2, 3]	2.67 ± 0.52; 3 [2, 3]	0.024
ASA missing	591/882 (67%)	10741/13493 (79.6%)	**-0.285**	527/786 (67%)	534/786 (67.9%)	-0.019
Diabetes	602/882 (68.3%)	2399/13493 (17.8%)	**1.019**	506/786 (64.4%)	485/786 (61.7%)	0.055
Insulin use	195/882 (22.1%)	189/13493 (1.4%)	**0.643**	156/786 (19.8%)	108/786 (13.7%)	0.163
Non-insulin diabetic drugs, count	1.5 ± 1.16; 2 [0, 2]	0.21 ± 0.5; 0 [0, 0]	**1.453**	1.38 ± 1.16; 2 [0, 2]	1.18 ± 1.05; 1 [0, 2]	0.188
Chronic medications, count	6.62 ± 3.11; 6 [4, 9]	3.62 ± 2.81; 3 [1, 5]	**1.013**	6.46 ± 3.16; 6 [4, 8.75]	6.26 ± 3.22; 6 [4, 8]	0.062
Chronic opioid use	53/882 (6%)	599/13493 (4.4%)	0.071	49/786 (6.2%)	42/786 (5.3%)	0.038
Pain modulator use	54/882 (6.1%)	466/13493 (3.5%)	0.125	45/786 (5.7%)	37/786 (4.7%)	0.046
Psychiatric medication use	196/882 (22.2%)	2698/13493 (20%)	0.055	177/786 (22.5%)	177/786 (22.5%)	0
NSAID use	12/882 (1.4%)	113/13493 (0.8%)	0.05	10/786 (1.3%)	12/786 (1.5%)	-0.022
Male sex	315/882 (35.7%)	4885/13493 (36.2%)	-0.01	278/786 (35.4%)	259/786 (33.0%)	0.051
Knee arthroplasty	624/882 (70.7%)	8438/13493 (62.5%)	0.175	551/786 (70.1%)	551/786 (70.1%)	0
Revision surgery	7/882 (0.8%)	148/13493 (1.1%)	-0.031	5/786 (0.6%)	9/786 (1.1%)	-0.054

Propensity-score model discrimination for GLP-1 RA exposure: AUC = 0.84; 1:1 matching yielded 786 matched pairs.

Values are mean ± SD or n/N (%). Continuous variables also show median [IQR]. SMD, standardized mean difference; bold values indicate meaningful imbalance between groups (|SMD| > 0.2).

BMI and ASA summaries are based on available data; missingness is reported separately.

ASA, American Society of Anesthesiologists physical status; BMI, body mass index; NSAID, nonsteroidal anti-inflammatory drug.

**Figure 2:**
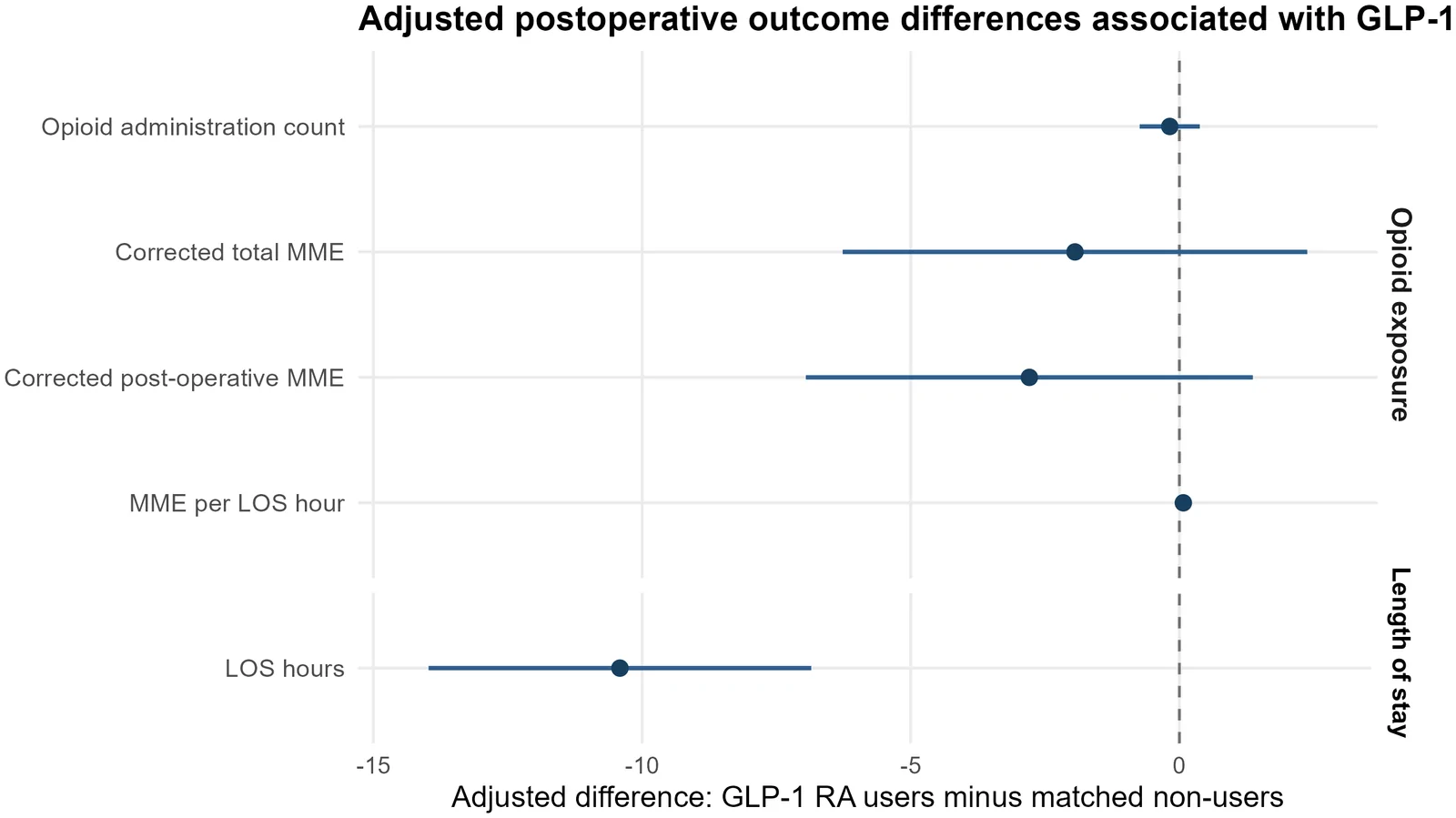
Forest plot showing adjusted differences for GLP-1receptor agonist users compared with matched non-users. Points represent adjusted estimates and horizontal lines represent 95% confidence intervals. Negative values indicate lower values among GLP-1 RA users. Estimates are shown on their original outcome scales. LOS, length of stay; MME, morphine milligram equivalents.

Main inpatient opioid and recovery outcomes are summarized in **Table 2** and adjusted between-group estimates are shown in **Figure 2**. In crude full-cohort comparisons, GLP-1 RA users appeared to have higher inpatient opioid exposure than non-users despite shorter length of stay. This pattern was attenuated after propensity-score matching, suggesting substantial confounding by baseline clinical differences.

**Table 2:** Main opioid and recovery outcomes.

Outcome	Analytic cohort crude difference	Matched unadjusted difference	Main adjusted difference	95% CI	p-value	Interpretation
Opioid administration count	1.22	-0.17	-0.18	-0.74 to 0.38	0.53	No significant difference
Corrected total MME	8.55	-1.66	-1.94	-6.27 to 2.38	0.378	No significant difference
Corrected post-operative MME	7.57	-2.51	-2.79	-6.95 to 1.37	0.188	No significant difference
MME per LOS hour	0.14	0.07	0.07	-0.00 to 0.15	0.0591	Borderline/not clearly significant
LOS hours	-3.51	-9.91	-10.41	-13.97 to -6.85	<0.001	Shorter in GLP-1 RA
LOS days	-0.15	-0.42	-0.44	-0.59 to -0.29	<0.001	Shorter in GLP-1 RA

Indicate lower values among GLP-1 RA users.

Main adjusted models used the fixed matched cohort and the final approved covariate framework.

LOS, length of stay; MME, morphine milligram equivalents.

In adjusted analyses of the matched cohort, GLP-1 RA use was not significantly associated with lower inpatient opioid administration use. The adjusted difference was −0.18 administrations among GLP-1 RA users compared with matched non-users (95% CI, −0.74 to 0.38; p=0.53). GLP-1 RA use was also not significantly associated with corrected total MME, with an adjusted difference of −1.94 MME (95% CI, −6.27 to 2.38; p=0.38), or corrected postoperative MME, with an adjusted difference of −2.79 MME (95% CI, −6.95 to 1.37; p=0.19).

When inpatient opioid exposure was expressed relative to hospitalization time, GLP-1 RA users had a slightly higher inpatient opioid use rate than matched non-users, although the difference was small and did not reach statistical significance. The adjusted difference was +0.073 MME per hospital hour, equivalent to approximately 1.8 MME per 24 hours, with a 95% CI crossing zero (−0.003 to 0.149; p=0.059). This small difference is unlikely to be clinically meaningful and should be interpreted in the context of shorter hospitalization among GLP-1 RA users.

GLP-1 RA use was consistently associated with shorter hospitalization before and after matching and adjustment. In the adjusted matched-cohort analysis, length of stay was 10.41 hours shorter among GLP-1 RA users compared with matched non-users (95% CI, −13.97 to −6.85; p<0.001), corresponding to approximately 0.44 fewer hospital days (95% CI, −0.59 to −0.29; p<0.001).

**Figure 3:**
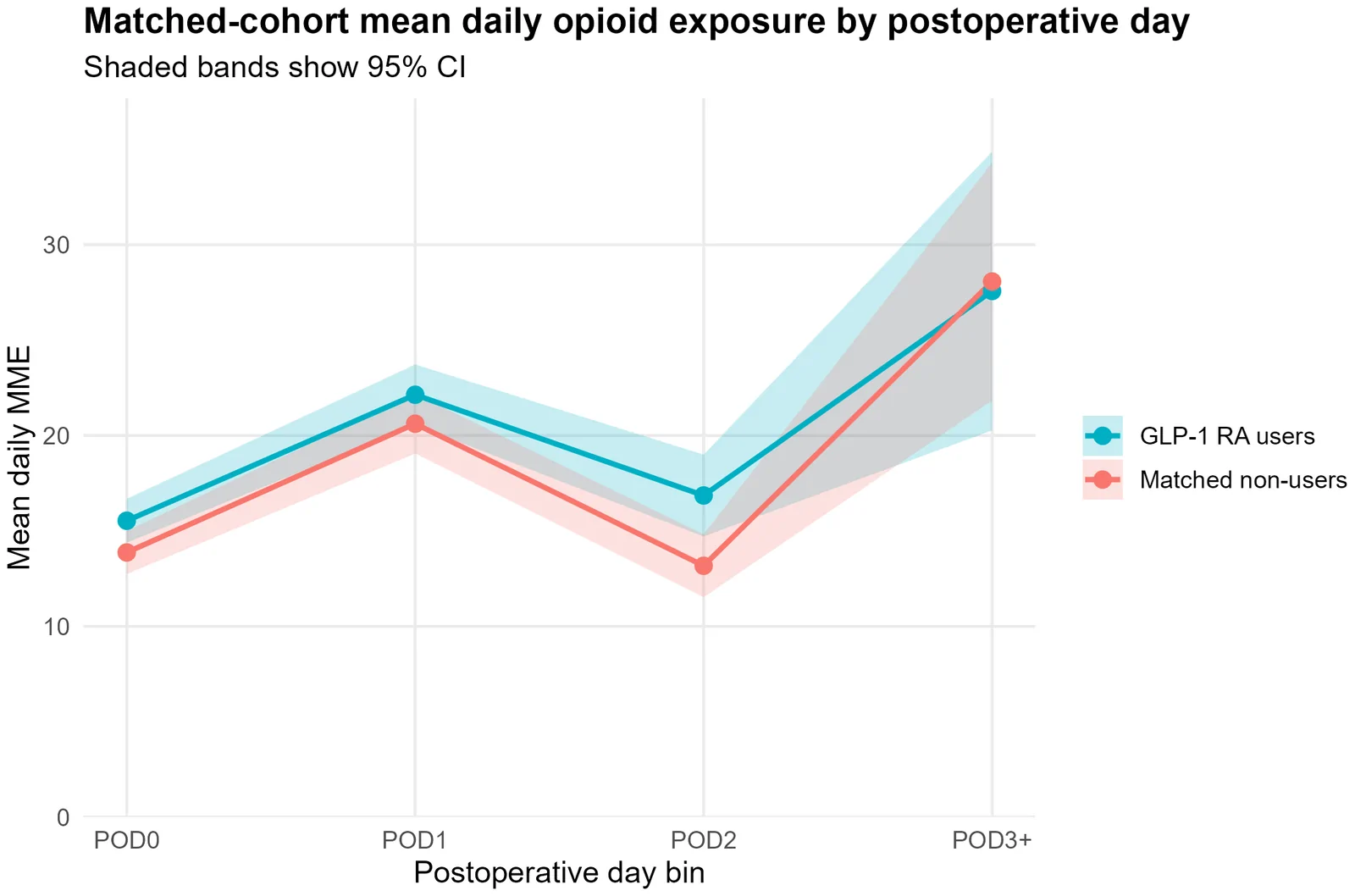
Matched-cohort daily opioid exposure by postoperative day. Mean daily opioid exposure is shown by postoperative day bin among GLP-1 receptor agonist users and matched non-users. Points and lines represent mean daily MME. and shaded bands represent 95% confidence intervals.POD3+ includes postoperative day 3 and alter. MME, morphine milligram equivalents; POD, postoperative day.

The POD-binned matched mean daily MME trajectory is shown in **Figure 3**, with full POD-binned opioid and pain trajectory outcomes provided in **Supplementary Table 2**. Postoperative day-binned inpatient opioid trajectories were examined descriptively across POD0, POD1, POD2, and POD3+, with POD3+ including postoperative day 3 and later among patients who remained hospitalized. The trajectory analysis did not demonstrate a consistent postoperative inpatient opioid-sparing pattern among GLP-1 RA users, consistent with the adjusted main opioid analyses. Because later POD bins include only patients who remained hospitalized, these comparisons were interpreted descriptively rather than as independent treatment-effect estimates.

In exploratory adjusted matched-cohort analyses, GLP-1 RA use was not significantly associated with an average difference in VAS score. The adjusted difference in average VAS was −0.094 points among GLP-1 RA users compared with matched non-users (95% CI, −0.221 to 0.034; p=0.15)., Similarly, in this exploratory analysis, the difference in maximum VAS between groups did not reach statistical significance, with an adjusted difference of approximately −0.45 points (95% CI, approximately −0.92 to 0.01; p≈0.056). The occurrence of any VAS score ≧7 was not significantly different between groups. Exploratory pain outcomes are summarized in **Supplementary Table 3**.

A subgroup analysis evaluating the specific common types of GLP-1 RAs administered revealed no significant differences in clinical outcomes between the groups. A detailed comparison across the various GLP-1 RA agents is presented in **Supplementary Figure 1**.

Finally, healthcare coverage was well balanced in the matched cohort, with absolute standardized mean differences below 0.10 across coverage categories. In a sensitivity analysis, additional adjustment for healthcare coverage produced estimates nearly identical to the primary results and did not change the direction or statistical significance of any association (**Supplementary Table 3**).

## Discussion

In this large retrospective cohort of patients undergoing hip or knee arthroplasty, chronic preoperative GLP-1 RA use was not associated with reduced inpatient opioid requirements after propensity-score matching and adjustment. GLP-1 RA users did not have significantly lower inpatient opioid administration use, or corrected postoperative MME compared with matched non-users. When inpatient opioid exposure was normalized to hospitalization time, GLP-1 RA users had a slightly higher MME rate per hospital day, although this difference was small, did not reach statistical significance, and is unlikely to be clinically meaningful.

In real-world perioperative setting, acute inpatient opioid requirements reflect not only individual pain intensity but also highly standardized care pathways. Postoperative opioid delivery is strongly driven by institutional analgesia protocols, nursing medication administration practices, anesthesia techniques, early mobilization targets, and discharge criteria. The overriding influence of these structured clinical pathways may mask subtle differences in individual pain perception, potentially explaining why the biologically plausible anti-inflammatory and antinociceptive properties of GLP-1 RAs did not translate into a measurable reduction in acute inpatient opioid administration.

In interpreting our findings, it is critical to distinguish acute inpatient opioid administration following total joint arthroplasty from chronic opioid consumption in osteoarthritis more broadly. Our study specifically evaluated the former; consequently, we did not assess outpatient opioid utilization, or long-term opioid dependence.

Consistent with the opioid findings, exploratory pain analyses did not demonstrate a clear association between GLP-1 RA use and improved early postoperative pain control; specifically, differences in maximum and average VAS scores did not reach statistical significance in these exploratory assessments.

The absence of an acute opioid-sparing effect aligns with emerging evidence from other surgical populations. Pennycuff et al. recently reported similar findings in a propensity-matched cohort of 486 oncology patients, where GLP-1 RA use was not associated with clinically meaningful reductions in perioperative opioid consumption or pain scores in the immediate postoperative period (16). A recent scoping review of 35 studies across orthopedic procedures also found that while GLP-1 RA use was associated with favorable metabolic and infectious outcomes, pain and opioid consumption showed inconsistent effects when measured (20).

Beyond the overriding influence of standardized care pathways, several other explanations may account for the lack of an acute opioid-sparing effect. First, the magnitude of surgical trauma, inflammatory activation, and nociceptive input following major joint arthroplasty may overwhelm any baseline anti-inflammatory or antinociceptive effects of chronic GLP-1 RA therapy (6, 9). Second, the potential anti-addictive properties of GLP-1 RAs, mediated through modulation of dopaminergic reward pathways, may be more relevant to voluntary, long-term opioid use patterns rather than acute inpatient administration (21). Third, it is possible that certain exposure patterns not measured in this cohort (e.g., longer use duration, specific indications) could produce measurable effects that were undetectable in this heterogeneous group. Finally, body composition changes associated with GLP-1 RA therapy may have complex and opposing effects on postoperative recovery. While weight loss and improved metabolic parameters may be beneficial, the concurrent loss of lean body mass could impair functional recovery and mobility. The net effect of these competing influences on acute pain and opioid requirements remains unclear.

In contrast, GLP-1 RA use was consistently associated with shorter hospitalization, both before and after matching and adjustment. After propensity-score matching and adjustment, GLP-1 RA users had approximately 10.4 fewer hospital hours (approximately 0.44 days) compared with matched non-users. This finding aligns with a 2026 scoping review reporting that GLP-1 RA exposure was generally associated with shorter lengths of stay in hip and knee arthroplasty cohorts, along with lower 90-day readmissions and reduced periprosthetic joint infection rates (20). Similarly, a large multicenter study found active GLP-1 RA prescriptions associated with reduced 30-day readmissions (22).

However, length of stay after arthroplasty is strongly influenced by non-biological factors that were not measured in this study. We emphasize that this finding must be interpreted strictly as hypothesis-generating rather than evidence of causal benefit. GLP-1 RA use may be a marker for patients engaged in more intensive preoperative metabolic care, weight-management programs, or multidisciplinary preoperative optimization pathways, reflecting inherently higher patient motivation and health literacy. Furthermore, length of stay depends heavily on unmeasured factors including socioeconomic status, home support systems, rehabilitation availability, and surgeon- or institution-specific discharge practices (2, 3, 5).

To partially address potential confounding related to healthcare access and financing, we performed a sensitivity analysis additionally adjusting for healthcare coverage category. The resulting estimates were nearly identical to the primary findings and did not change the direction or statistical significance of any association. However, healthcare coverage is only a limited proxy and does not directly capture socioeconomic status or discharge disposition. Prospective studies with detailed data on discharge disposition, post-discharge care utilization, functional recovery trajectories, and patient-reported outcomes are needed to clarify whether GLP-1 RA therapy genuinely facilitates earlier recovery or whether the observed association reflects unmeasured confounding.

This study has several limitations. First, the retrospective design limits causal inference. Second, although propensity-score matching substantially improved measured covariate balance, residual imbalances in BMI and diabetes-treatment variables remained. While we accounted for these through additional covariate adjustment in our final outcome models, residual metabolic differences between the groups cannot be entirely ruled out and may still have influenced postoperative recovery and discharge timing. Furthermore, we lacked detailed data on body composition, sarcopenia, frailty, and specific perioperative care pathways, which are particularly relevant to the observed association with shorter LOS. Third, the study was conducted at an elective surgical center, which may limit generalizability to other practice settings with different discharge practices and patient demographics. Finally, the analysis was restricted to inpatient outcomes and did not include long-term functional recovery, complications, or readmissions.

In conclusion, chronic preoperative GLP-1 RA use was not associated with reduced inpatient opioid exposure or clearly improved early postoperative pain control after hip or knee arthroplasty. GLP-1 RA use was, however, consistently associated with shorter hospitalization. Prospective studies with standardized pain assessments, discharge-disposition information, and post-discharge opioid use follow-up are needed to clarify whether GLP-1 RA therapy influences recovery pathways after arthroplasty.

## Data Availability

The original contributions presented in the study are included in the article/**Supplementary Material**. Further inquiries can be directed to the corresponding author.
