# Supplementary material for: Prehospital glucagon-like peptide-1 receptor agonists are not associated with reduced inpatient opioid exposure but with shorter hospital length of stay after total joint arthroplasty
Source: Front Endocrinol (Lausanne). 2026 Aug 21;17:1918370. doi: 10.3389/fendo.2026.1918370 (PMC13541510; doi:10.3389/fendo.2026.1918370)
Supplement: Supplementary file 1 [file Image1.pdf]

Supplementary Figure 1.
Glp-1 receptor agonist agent distribution and postoperative outcomes in the matched cohort.

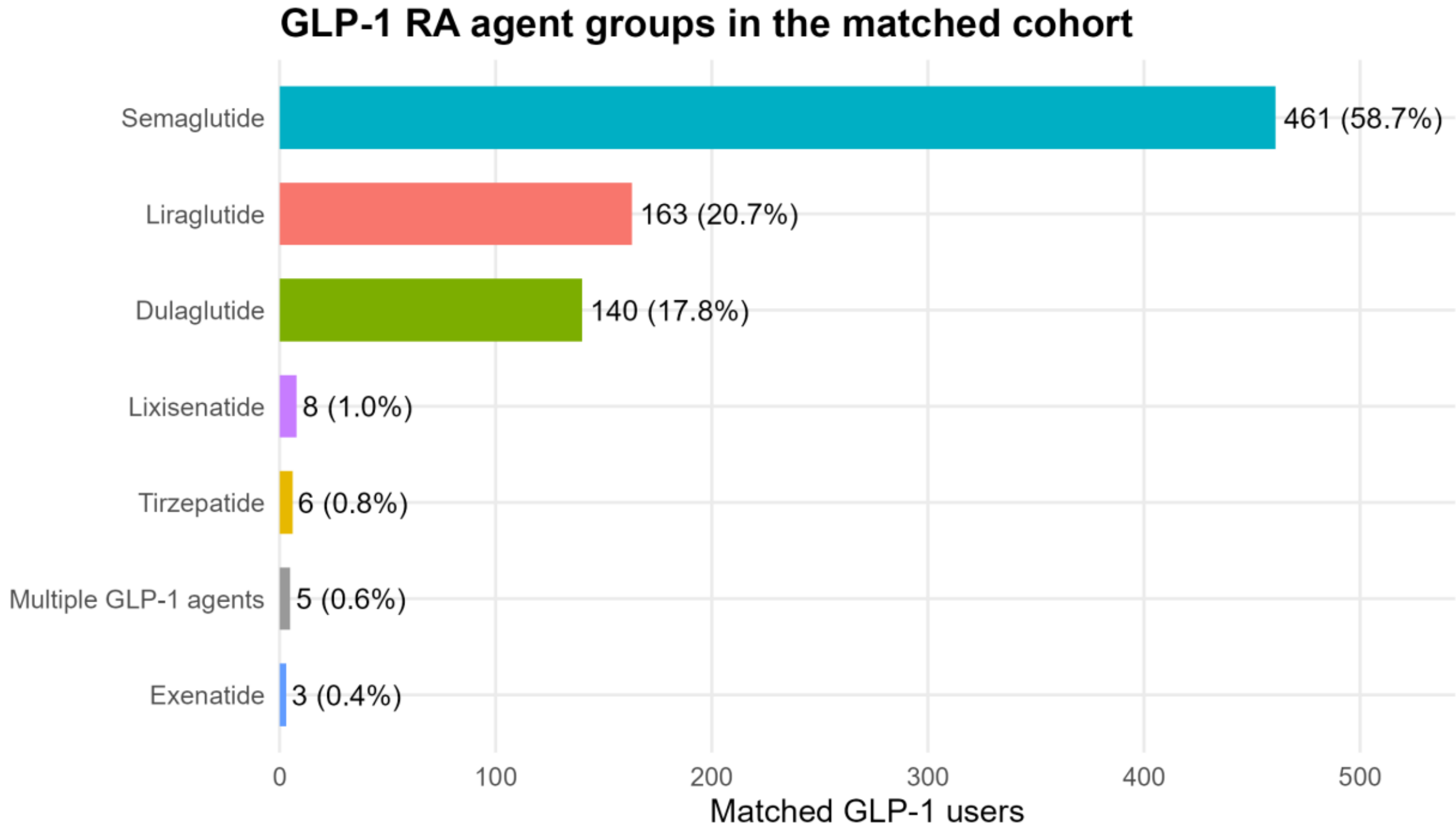

(A) Distribution of GLP-1 receptor agonist agents among matched GLP-1 RA users. Semaglutide was the most common agent, while other agents represented small subgroups.

(B) Descriptive comparison of postoperative opioid use, length of stay, and pain outcomes across the three main GLP-1RA agents. Points represent interquartile ranges.

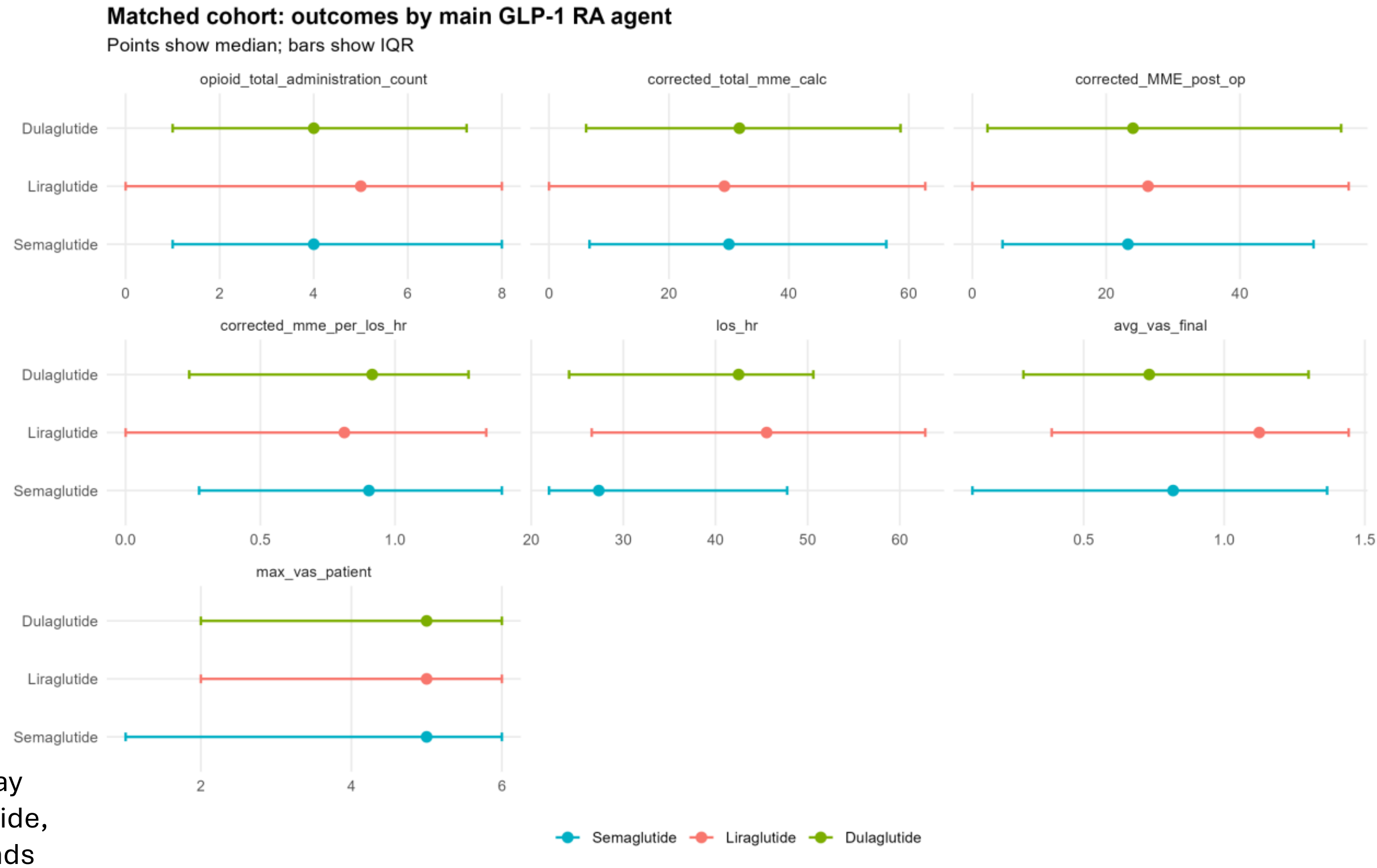

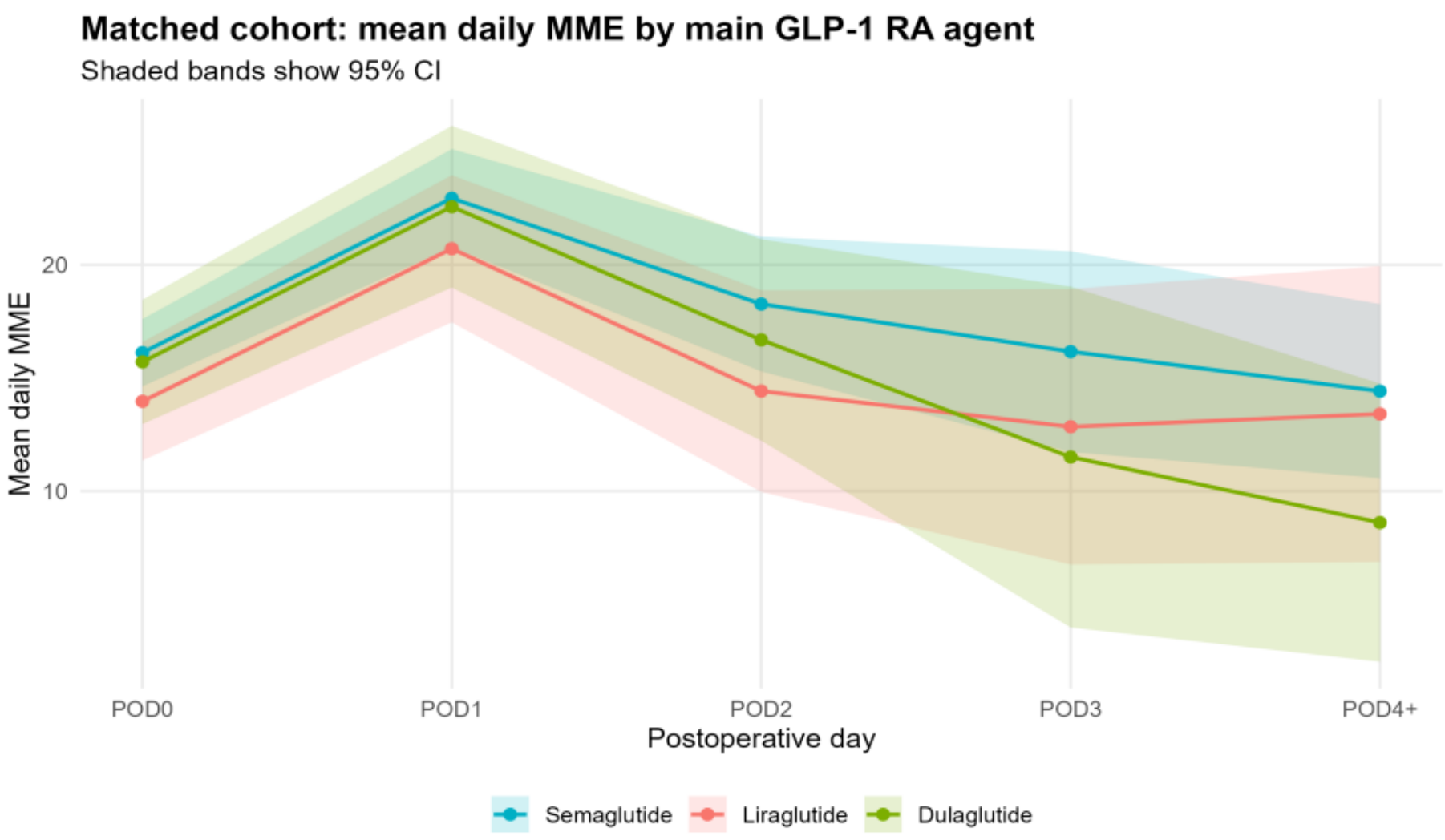

(C) Mean daily morphine milligram equivalents (MME) by postoperative day among patients treated with semaglutide, liraglutide, or dulaglutide. Shaded bands represent 95% confidence intervals.
